# Alpha-Lipoic Acid Preserves Testicular Integrity Under 2.45 GHz Electromagnetic Radiation by Restoring Redox and Inflammatory Balance

**DOI:** 10.3390/biomedicines13123089

**Published:** 2025-12-15

**Authors:** Tahir Cakir, Seda Keskin, Kenan Yildizhan, Mehmet Hafit Bayir, Fikret Altindag, Erbil Karaman

**Affiliations:** 1 Department of Biophysics, Faculty of Medicine, Van Yuzuncu Yil University, Van 65100, Turkey; tahircakir@yyu.edu.tr (T.C.); kenanyildizhan@yyu.edu.tr (K.Y.); 2 Department of Histology and Embryology, Faculty of Medicine, Van Yuzuncu Yil University, Van 65100, Turkey; sedakeskin@yyu.edu.tr (S.K.); mehmethafitbayir@yyu.edu.tr (M.H.B.); fikretaltindag@yyu.edu.tr (F.A.); 3 Department of Gynecology and Obstetrics, Faculty of Medicine, Van Yuzuncu Yil University, Van 65100, Turkey

**Keywords:** alpha-lipoic acid, 2.45 GHz electromagnetic radiation, testicular damage, oxidative stress, inflammation

## Abstract

**Background/Objective:** Electromagnetic radiation (EMR) from wireless technologies has raised concerns about male reproductive health. We aimed to evaluate the protective role of alpha-lipoic acid (ALA), a potent antioxidant, against testicular alterations induced by 2.45 GHz EMR. **Methods:** Twenty-eight adult male rats were randomly divided into four groups: control, EMR, ALA, and ALA+EMR. Animals in the EMR and ALA+EMR groups were exposed to EMR for 2 h/day for 1 month. Testicular tissues were examined histologically, stereologically, and immunohistochemically, while serum samples were analysed biochemically. **Results:** EMR exposure caused marked structural damage, including disruption of seminiferous tubule architecture, increased collagen deposition, and expansion of tubular and interstitial volumes. These pathological changes were primarily prevented in the ALA+EMR group. Immunohistochemical analyses revealed increased IL-6 and TNF-α expression following EMR exposure, whereas ALA supplementation significantly reduced these inflammatory markers and restored AR, ZO-1, and ZO-2 expression. Biochemically, EMR reduced antioxidant enzyme activities (SOD, GSH, GPx) and elevated MDA levels, indicating oxidative stress; these parameters were reversed by ALA treatment. **Conclusions:** Collectively, our findings demonstrate that 2.45 GHz EMR induces oxidative stress, inflammation, and testicular injury, while ALA provides significant protection. These results highlight the therapeutic potential of ALA as a protective agent against EMR-related reproductive toxicity and infertility risk.

## 1. Introduction

Today, exposure to electromagnetic radiation (EMR) has become a significant public health concern due to the rapid proliferation of wireless communication technologies, mobile phones, Wi-Fi devices, and other EMR sources ubiquitous in daily life. Wireless communication systems operating at 2.45 GHz have been the subject of intensive research in recent years due to their potentially harmful effects on biological tissues. Numerous studies have demonstrated that prolonged, high-frequency exposure to EMR can induce oxidative stress, inflammation, DNA damage, and cellular dysfunction. In this context, the male reproductive system, particularly testicular tissue, is among the most sensitive organs to EMR-induced biological stresses [1,2,3].

The testes are highly sensitive to environmental stressors due to their high metabolic activity, the continuous cell division and differentiation that occur during spermatogenesis, and their specialised anatomical structures, such as the blood-testis barrier. Structural changes in testicular tissue resulting from EMR exposure have been reported, including morphological alterations, irregularities in the seminiferous tubule structure, collagen accumulation, and expansion of the interstitial space [2,4]. Furthermore, increased inflammatory cytokines (IL-6, TNF-α), suppression of antioxidant defence systems (SOD, GSH, GPx), and elevated lipid peroxidation products (MDA) reveal the adverse effects of EMR on reproductive health at the molecular level. This situation is considered a mechanism that poses a direct threat to male fertility [5,6].

In recent years, considerable interest has arisen in natural antioxidant compounds to reduce EMR-related damage at the cellular and tissue levels. Several animal studies have previously investigated different antioxidant or cytoprotective agents against EMR-induced oxidative and inflammatory damage, including coenzyme Q10, vitamin C, gallic acid, and melatonin, all of which have demonstrated varying degrees of tissue protection by modulating redox balance or inflammatory signalling pathways [6,7,8,9]. Despite these studies, ALA’s protective potential against 2.45 GHz EMR-induced testicular injury has not been comprehensively evaluated. Therefore, testing ALA specifically in the EMR model addresses an unmet need in the current literature and provides new insights into whether its potent antioxidant–anti-inflammatory properties can mitigate EMR-related testicular damage.

Among these compounds, alpha-lipoic acid (ALA) is a potent endogenous antioxidant that is soluble in both lipophilic and hydrophilic environments. ALA can directly scavenge free radicals and enhance the activity of endogenous antioxidant enzymes, such as glutathione, superoxide dismutase, and catalase [10,11]. Furthermore, ALA is widely recognised for its antioxidant and cytoprotective properties, and previous studies have demonstrated that ALA can attenuate oxidative stress and inflammatory responses in various experimental models [12,13,14,15,16,17]. Due to these properties, ALA is considered a therapeutic candidate in many pathological conditions where oxidative stress and inflammation play a critical role, including neurodegenerative diseases, diabetic complications, cardiovascular diseases, and infertility [11,16,18].

Although several antioxidants, such as coenzyme Q10, vitamin C, gallic acid, and melatonin, have been shown to offer partial protection against EMR-induced testicular oxidative injury [6,7,8,9], the potential protective role of ALA—a broad-spectrum antioxidant and anti-inflammatory molecule—remains insufficiently explored. Therefore, the present study addresses this gap explicitly by evaluating the histological and biochemical protective effects of ALA against 2.45 GHz EMR-induced testicular damage. Given that our experimental model involves EMR-induced inflammation-driven oxidative damage, the inclusion of ALA provides a mechanistically relevant therapeutic candidate that could counteract both oxidative and inflammatory components of the pathology. Therefore, ALA was selected owing to its well-established cytoprotective profile and its documented efficacy in similar inflammatory injury models. Although the current literature suggests that antioxidant strategies can reduce EMR-induced damage to testicular tissue, studies investigating the protective role of ALA in this context are limited. The conceptual framework of this study centres on the emerging role of ALA as a systemic redox–endocrine modulator. Beyond its well-established antioxidant properties, ALA influences mitochondrial redox balance, regulates steroidogenic enzyme activity, and modulates hormonal responses associated with inflammation-induced tissue injury. These integrated redox–endocrine interactions provide a mechanistic basis that distinguishes ALA from conventional antioxidant interventions and highlight its potential to modulate systemic oxidative and endocrine dysregulation. Accordingly, this study investigates ALA within a broader mechanistic context, aiming to elucidate its coordinated effects on oxidative stress, endocrine signalling, and tissue integrity. Data on how ALA modulates the structural integrity, barrier proteins (ZO-1, ZO-2), androgen receptor (AR) expression, the inflammatory response, and biochemical parameters in testicular tissue remain insufficient. Therefore, this study systematically examined the histological, immunohistochemical, and biochemical changes induced by 2.45 GHz EMR exposure in testicular tissue and investigated the potential protective effects of ALA. Consequently, this study aims to make an essential contribution to better understanding the harmful effects of EMR on male reproductive health and to reveal the therapeutic potential of ALA. The findings may not only shed light on the mechanistic aspects of EMR bioeffects but also provide a scientific basis for developing new antioxidant-based strategies.

## 2. Materials and Methods

### 2.1. Animals and Experimental Design

A total of 28 adult male *Wistar-albino* rats (150–250 g) were used in this study. The animals were obtained from the Van Yuzuncu Yil University Laboratory Animal Research Unit. All procedures were performed in accordance with the National Institutes of Health (NIH) Guide for the Care and Use of Laboratory Animals and were approved by the Van Yuzuncu Yil University Institutional Animal Care and Use Committee (Approval No: 2021/06-42). During the experiment, the animals were housed in stainless steel cages with a 12-hour light/dark cycle, at 20–22 °C and 50–55% relative humidity. All animals had unlimited access to standard pellet feed and tap water.

Animals were randomly allocated to the experimental groups using simple randomisation. Animals were randomly assigned to four groups (*n* = 7/group): Control group (CON), in which no treatment was administered, and animals were maintained under standard conditions only. Electromagnetic radiation (EMR) group: Exposed to 2.45 GHz electromagnetic radiation for 2 h daily (10:00–12:00) for 30 days [9]. Alpha-lipoic acid (ALA) group: Administered α-lipoic acid (30 mg/kg/day; Solgar®, Leonia, NJ, USA) orally via gavage for 30 days without 2.45 GHz EMR exposure [19]. The ALA dose (30 mg/kg) was selected based on previous in vivo studies in rats that demonstrated potent antioxidant and anti-inflammatory effects at this concentration without observable toxicity [18,19,20,21]. ALA+EMR (treatment) group: Following the same ALA dose, the animals were exposed to 2.45 GHz EMR (12:00–14:00) for 2 h daily for 30 days. ALA was administered by oral gavage 1 hour before each EMR exposure to ensure adequate systemic absorption. This timing was chosen to ensure that systemic ALA levels were elevated during the EMR exposure period. The same gavage schedule was applied for the ALA-only group. All administrations and exposures were performed at consistent daily time points to minimise circadian variation.

Then, 2.45 GHz EMR exposure was conducted in a specially designed chamber, and the ambient radiation was verified using a broadband EMR metre before the animals were placed inside. The control and ALA-only groups were not exposed to EMR. On the 30th day of the experiment, all animals were anaesthetised with ketamine hydrochloride (50 mg/kg) and xylazine (5 mg/kg), and a cardiac dissection procedure was performed after deep anaesthesia was achieved.

### 2.2. EMR Exposure System

In the experimental study, a specialised exposure chamber was constructed within the Van Yuzuncu Yil University Laboratory for the Production and Research of Experimental Animals, dedicated solely to this purpose, to conduct electromagnetic radiation (EMR) applications. Rats belonging to the EMR and ALA+EMR groups were exposed to a specially designed EMR system located in this chamber. Other experimental groups were maintained in separate chambers under constant animal-care conditions. Rats were exposed to EMR at 2.45 GHz for 2 h per day for 30 consecutive days [22]. Exposures were conducted in plexiglass cages measuring 30 × 42 × 50 cm, each designed to house 6–8 rats. The cages were positioned to maintain a 39 cm distance between the antenna and the animals [23].

The EMR was generated by a voltage-controlled oscillator (VCO), amplified by a wideband RF amplifier, and transmitted to a half-wave monopole antenna via coaxial cable. This antenna, developed at the Antenna Laboratory of Karadeniz Technical University, was placed 39 cm above the cage floor. The field strength was regularly recorded both when the animals were in the cage and in an empty environment using a broadband EMR metre capable of measuring in the 100 kHz–3 GHz range [24]. The microwave generator’s output was validated using a calibrated spectrum analyser and power metre, confirming a delivered microwave power of approximately 24 dBm (≈252 mW). To characterise the physical exposure conditions, field intensity measurements were performed at the level of the animal cages. The distance between the antenna and the cage centre was fixed at 39 cm, resulting in an estimated power density of ~0.6 mW/cm^2^ at the animal location. The whole-body specific absorption rate (SAR) was calculated using standard dosimetric equations (SAR = σE^2^/ρ), yielding an estimated SAR of 0.00588 W/g. These values fall within the reported range for low-level 2.45 GHz experimental exposures in rodent studies, thereby enabling appropriate comparison with previously published EMR research.

Radiation homogeneity was controlled theoretically and experimentally using a printed monopole antenna with 1 dB gain, optimised with the IE3D simulation tool. Additionally, plexiglass plates placed between the cage structures balanced the distribution of the exposure area, enhancing EMR homogeneity.

At 2.45 GHz exposure (λ ≈ 12.24 cm), the antenna-to-cage distance of 39 cm ensured far-field conditions. Local SAR values were calculated using electric field strengths (E) measured at nine predetermined points on the cage floor. Power density was derived as S = E^2^/Z_0_, and SAR was computed using SAR = σE^2^/(2ρ), with tissue conductivity (σ = 1.7 S/m) and density (ρ = 1040 kg/m^3^) adopted for muscle-equivalent tissue. The mean local SAR across the nine measurement points was 0.00588 W/kg (SD = 0.00153 W/kg; range: 0.00425–0.00890 W/kg). Since the duty cycle was 1.0, the time-averaged SAR remained identical. Using conservative whole-body absorption parameters (η_abs = 0.5, A_proj = 35 cm^2^), the whole-body SAR was estimated to fall within the 0.000134–0.000223 W/kg range.

#### Calibration and Field Homogeneity Assessment of the EMR System

The electromagnetic exposure system was calibrated prior to the experiments using a spectrum analyser and a broadband electromagnetic field metre (100 kHz–3 GHz). The antenna input power was confirmed to be approximately 24 dBm (≈252 mW). Electric field strength was measured at nine distinct positions within the exposure cage to verify field homogeneity across the animal holding area. The spatial variation in field intensity remained minimal, confirming that the animals were exposed to a uniform electromagnetic field. Throughout the exposure period, the ambient temperature inside the animal cage was continuously monitored, and no measurable increase in temperature was observed. Given the system’s low power density (0.0191 W/m^2^) and the calculated specific absorption rate (SAR ≈ 0.006 W/kg), thermal effects were not expected and were experimentally excluded.

To exclude possible thermal effects during EMR exposure, ambient and cage-level temperatures were routinely monitored using a calibrated digital thermometer placed adjacent to the exposure chamber. Measurements were recorded before, during, and after each EMR session.

### 2.3. Histological Procedure

#### 2.3.1. Hematoxylin–Eosin (H&E) Staining

Tissue samples obtained from the right testicles were fixed in 10% neutral buffered formalin. Following fixation, the tissues were dehydrated in a series of ethanol concentrations and then cleared in xylene. Five-µm-thick sections were prepared from the paraffin-embedded samples using a rotary microtome (Leica, Wetzlar, Germany). H&E staining was used for histopathological examination to assess the overall tissue architecture. Following staining, the preparations were examined at different magnifications (×100 and ×200) using a Nikon Eclipse Ni light microscope and a Nikon DS-Fi2 digital camera system [25]. Two independent observers performed histopathological evaluations on multiple randomly selected sections from each animal.

#### 2.3.2. Johnsen’s Scoring System

Tissue samples obtained from the left testis of each experimental animal were examined to evaluate the spermatogenic activity of the seminiferous tubule structure. Within this scope, cross-sections of between 20 and 30 seminiferous tubules randomly selected from each section were analysed. This approach enabled comparative assessments of seminiferous tubule organisation, cellular differentiation stages, and spermatogenic capacity between experimental groups, thereby facilitating objective determination of the effects of electromagnetic radiation exposure and ALA administration on testicular histology. The analyses were based on the scoring system defined by Johnsen, which was later modified for widespread use [26]. Each tubule was evaluated on a scale from 1 to 10 according to the established criteria. Johnsen’s Scoring was performed blinded by an investigator unaware of the group assignments. Since the seminiferous epithelial phase was not standardised during morphometric measurements in this study, determining the spermatogenesis index is critical for distinguishing the phase of each tubule. The Johnsen score quantitatively reflects the functional integrity of spermatogenesis, with higher scores indicating normal spermatogenesis and lower scores indicating impaired or insufficient spermatogenesis [27]. The final Johnsen score for each tissue was calculated using the following formula [26,27,28]: Final Johnsen score, $\mu =\frac{\sum (x)(y)}{z}$.

Note: *x*: a score of each seminiferous tubule; *y*: number of tubules with the assigned score; *z*: total number of evaluated tubules.

#### 2.3.3. Masson’s Trichrome (MT) Staining

MT staining was applied to determine fibrotic changes and collagen distribution in testicular tissue. The staining procedures were performed using an MT staining kit (Cat. No. 04-010802, Bio-Optica, Milan, Italy) according to the manufacturer’s protocol. The MT-stained sections were examined using a Nikon Eclipse Ni light microscope (Tokyo, Japan) and a Nikon DS-Fi2 camera system. All images were evaluated at 20× and 40× magnifications using NIS Elements F 4.00.00 software. The positive areas were quantified in at least five randomly selected heart sections using ImageJ (Fiji, Version 1.54i) [29].

#### 2.3.4. Immunohistochemical Staining

Testicular tissues from all experimental groups were fixed in 10% neutral buffered formalin for 72 h. Subsequently, five μm-thick paraffin sections, mounted on positively charged slides, were incubated overnight at 37 °C in an oven. The sections were placed in xylene for 5 min, three times, for deparaffinization, then in pure alcohol and 96% ethanol for 10 min each. To block endogenous peroxidase activity, they were incubated for 20 min in a 3% hydrogen peroxide solution prepared in methanol. The sections were then washed first with tap water and then with distilled water. For antigen retrieval, the sections were heated in a 300-watt microwave oven in citrate buffer for 20 min, then cooled to room temperature. Once they reached room temperature, the sections were washed three times for 5 min each in PBS, then incubated in blocking solution (Thermo Scientific, Waltham, MA, USA) for 10 min. For primary antibody incubation, the sections were incubated overnight at 4 °C with anti-androgen receptor (AR) (sc-7305), anti-proliferating cell nuclear antigen (PCNA) (F-2) (sc-25280), anti-IL-6 (sc-130326), anti-tumour necrosis factor-α (TNF-α) (52B83) (sc-52746), anti-zonula occludens-1 (ZO-1) (R40.76) (sc-33725), and anti-zonula occludens-1 (ZO-2) (E-3) (sc-515115) antibodies. After three washes with PBS (each for five min.), the sections were incubated with a biotinylated secondary antibody (Thermo Scientific, USA) for 30 min. After rewashing with PBS, the sample was incubated with streptavidin-peroxidase (Thermo Scientific) for 10 min. Sections developed with DAB chromogen (3,3′-diaminobenzidine) for 5–10 min were counterstained with Mayer’s hematoxylin after washing with distilled water. Finally, sections were sealed with Entellan. IHC staining procedures were performed using a standardised protocol [30]. Non-specific staining was verified by omitting the primary antibody, and all samples were processed simultaneously under identical conditions for reliable comparative evaluation. Stained preparations were imaged at 20× and 40× objective magnifications using a Nikon Eclipse Ni light microscope (Tokyo, Japan) and a Nikon DS-Fi2 digital camera system. Imaging and recording were performed using NIS Elements F 4.00.00 software (Nikon Imaging Software Solutions, Tokyo, Japan). IHC intensity measurements for markers were performed in a blinded manner by an investigator unaware of group assignments.

### 2.4. Histomorphometric Measurements on Testicular Tissue

The diameter of the seminiferous tubules and the height of the seminiferous tubule epithelium were measured using ImageJ (Fiji, Version 1.54i, NIH, Pike Bethesda, MD, USA). Collagen fibre density was assessed in MT-stained sections using ImageJ/Fiji across 10–15 regions per animal [25]. For immunohistochemical staining, the positive staining intensities for AR, PCNA, IL-6, TNF-α, ZO-1, and ZO-2 were quantified using ImageJ/Fiji. For each rat, 10 randomly selected regions of interest were evaluated across three non-overlapping sections. An average of 10–15 fields per animal was selected by random sampling [31]. All images were obtained using a Nikon Eclipse Ni light microscope equipped with a Nikon DS-Fi2 camera and analysed using NIS Elements F 4.00.00 software (Nikon Imaging, Japan).

### 2.5. Stereological Analysis

Stereological evaluation of the seminiferous tubules and interstitial area density was conducted using the Cavalieri principle [32,33]. From paraffin-embedded testicular tissues, an average of 10–12 sections per sample were obtained using a rotary microtome at a thickness of five µm. The first section was selected at random, followed by every 30th section thereafter. All sections were stained with H&E and mounted with Entellan. Volumes of the seminiferous tubules and interstitial area of each subject were calculated using the point counting technique based on the Cavalieri principle, in accordance with stereological methods. The formula used for testis volume calculations is as follows:


*Volume (V) = (a/p) × ∑P × t*

Note: V: the relevant volume, t: section thickness, a/p: the area represented by each point, ΣP: the total number of points falling on the section profile.

### 2.6. Biochemical Analysis

Blood and testis tissues were collected promptly after the experiment for biochemical evaluations. Blood samples were allowed to clot at room temperature, then centrifuged at 4 °C for 15 min at 1000× *g* to separate the serum, which was stored at −20 °C until the day of analysis. Testicular tissues were homogenised with phosphate buffer to produce 10% homogenates. After homogenization on ice at 12,000 rpm for 1–2 min, the homogenate was centrifuged at 5000 rpm for 30 min to obtain the supernatant. Testosterone and thyroid-stimulating hormone (TSH) levels were measured using a Roche diagnostic kit (Basel, Switzerland) on a Cobas 8000 HITACHI system. Superoxide dismutase (SOD) and malondialdehyde (MDA) levels in the supernatants were measured spectrophotometrically using commercial kits. SOD activity was assessed based on the inhibition of the conversion of superoxide radicals produced by the xanthine–xanthine oxidase system into the formazan dye 2-(4-iodophenyl)-3-(4-nitrophenol)-5-phenyltetrazolium chloride (INT). One SOD unit was defined as corresponding to a 50% inhibition of INT reduction. MDA levels were analysed according to a kit protocol that quantifies lipid peroxidation products via thiobarbituric acid reactive substances (TBARS). In addition, glutathione (GSH) and glutathione peroxidase (GPx) levels were evaluated in serum samples. GSH and GPx levels were determined using a biotin-labelled double-antibody sandwich ELISA method, and results were calculated in ng/mL. All biochemical parameters were compared between experimental groups using appropriate statistical tests.

### 2.7. Statistical Analysis

Statistical analyses were performed using GraphPad Prism 9.0 software (GraphPad Software, San Diego, CA, USA). Normality of data distribution was evaluated using the Shapiro–Wilk test, and homogeneity of variances was assessed using Levene’s test. When these assumptions were met, intergroup comparisons were conducted using one-way or two-way ANOVA followed by Tukey’s post hoc test. Immunohistochemical staining intensities quantified in ImageJ/Fiji were compared using unpaired Student’s *t*-tests under the assumption of normality. All quantitative data are presented as mean ± SEM, and error bars in all graphs represent SEM. GraphPad Prism reported statistical outputs as significance ranges rather than exact *p*-values. Results are reported using standard significance thresholds (* *p* < 0.05, ** *p* ≤ 0.01, *** *p* ≤ 0.001, and **** *p* ≤ 0.0001).

## 3. Results

### 3.1. Effects of 2.45 GHz EMR Exposure on Physiological Parameters in Rats

During the 30-day experimental period, no abnormalities in general appearance or behaviour were observed in any group, and no deaths occurred. No thermal changes were detected during EMR exposure (ΔT < 0.3 °C). Thus, all biological changes observed in the study are due to non-thermal EMR effects.

Body weight measurements taken on days 1, 15, and 30 of the experiment were evaluated. The average body weight percentage change on day 15 in the EMR group was found to be significantly higher than in the CON group (Supplementary Figure S1A, *** *p* < 0.001). However, the change on day 30 was not statistically significant. In the ALA+EMR group, this percentage change was found to be significantly reduced compared to the EMR group (Supplementary Figure S1A, *** *p* < 0.001). When water consumption was examined, the average daily water intake of rats in the EMR group exposed to 2.45 GHz EMR increased significantly compared with the control group (Supplementary Figure S1B, *** *p* < 0.001). In the ALA+EMR, this increase was found to be substantially lower than in the EMR group (Supplementary Figure S1B, *** *p* < 0.001). At the end of the experiment, the testicles were weighed to assess organ mass. In the EMR group, testicular weights were significantly elevated in comparison to the control group (Supplementary Figure S1C, * *p* < 0.05). In the ALA+EMR group, this increase was found to be reduced considerably compared to the EMR group (Supplementary Figure S1C, *** *p* ≤ 0.001).

### 3.2. ALA Attenuates 2.45 GHz EMR-Induced Histopathological Alterations in Testicular Tissue

**Figure 1:**
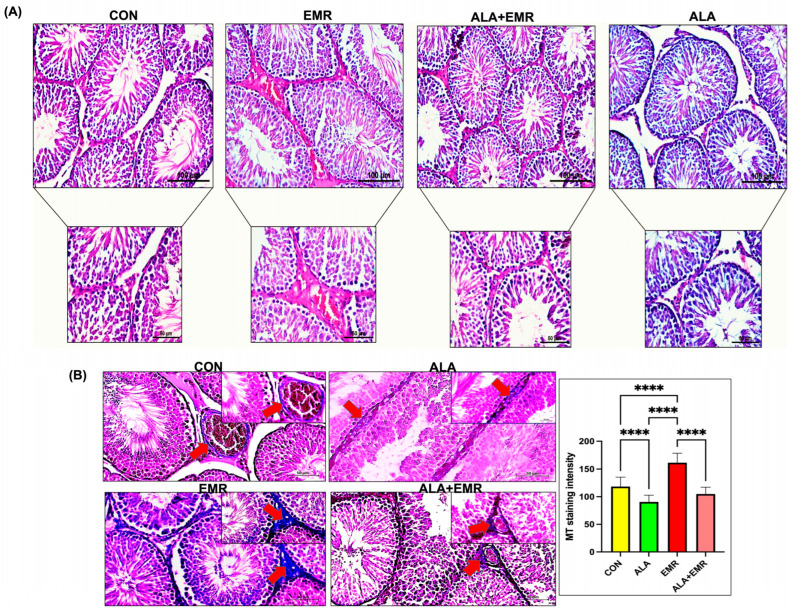
Histopathological assessment of testicular tissue following 2.45 GHz electromagnetic radiation (EMR) exposure and the protective effect of alpha-lipoic acid (ALA). (**A**) Representative hematoxylin and eosin (H&E)-stained sections from Control (CON), EMR, ALA, and ALA+EMR groups (H&E, Scale bar: 100 μm and 50 μm, Magnification: ×20 and ×40) (**B**) Masson Trichrome (MT) staining of testicular sections from all experimental groups. Representative MT-stained sections in the CON, ALA, and ALA+EMR (treatment) groups. Red arrows indicated collagen deposition (MT staining, Scale bar:20 μm and 50 μm, Magnification: ×20 and ×40). Quantitative analysis of MT staining intensity confirmed a significant increase in collagen content in the EMR group compared to all other groups. Data are presented as mean ± SEM (*n* = 7). Statistical significance was assessed using ANOVA with Tukey’s post hoc test or unpaired *t*-test where appropriate; **** *p* ≤ 0.0001.

Histopathological assessment indicated that the tunica albuginea, seminiferous tubules, interstitial connective tissue, and Leydig cells displayed normal histomorphology in the H&E-stained testicular sections exclusively from the CON, ALA, and ALA+EMR groups. The basal lamina of the seminiferous tubules was found to be regular, the germinal epithelium maintained its structural integrity, and the Sertoli cells and spermatogonia were found to be in a physiological configuration. No haemorrhage, inflammation, or degenerative lesions were detected in the CON, ALA and ALA+EMR groups (Figure 1A). In contrast, significant histopathological changes were observed in the EMR group. Localised epithelial detachment and reduction in epithelial thickness in the seminiferous tubule epithelium, separations in the basal lamina, significant losses in germ cells, oedema and congestion in the interstitial area, and vacuolisation in the seminiferous epithelium were observed (Figure 1A). Furthermore, a decrease in interstitial connective tissue was seen compared to the CON group. In the ALA+EMR group, these structural alterations were observed to be less severe. Despite persistent epithelial detachments, germ cell loss, interstitial oedema, and vacuolization observed in the EMR group, ALA administration markedly reduced lesion intensity (Figure 1A). Histochemical comparisons generally revealed that EMR exposure caused severe damage to testicular tissue. At the same time, ALA significantly mitigated EMR-related alterations at 2.45 GHz. It was concluded that CON and ALA-only groups maintained near-normal histological integrity and that ALA exhibited a pronounced tissue-protective effect against 2.45 GHz EMR. Table 1 summarises the histopathological results across the experimental groups.

### 3.3. ALA Attenuates 2.45 GHz EMR-Induced Fibrotic Remodelling in Testicular Tissue

MT staining was applied to evaluate collagen distribution in the interstitial connective tissue of the testicular tissues. In the CON and ALA-only groups, minimal accumulation of collagen fibres was observed in the tunica vasculosa layer, limited to the basal membranes of the seminiferous tubules and blood vessels (Figure 1B). In contrast, significant collagen accumulation was detected in the EMR group (Figure 1B, **** *p* < 0.0001). Dense, compact collagen bundles were observed in the interstitial areas and around the basement membrane. In the ALA+EMR group, a significant decrease in collagen accumulation was observed compared to the EMR group (Figure 1B, **** *p* < 0.0001). The collagen fibre distribution was assessed as returning to a more regular, finer structure, closer to that of the CONT group. Taken together, these findings indicate that 2.45 GHz EMR exposure increases the fibrotic response in the testicular interstitium, but ALA treatment significantly suppresses collagen accumulation.

### 3.4. Spermatogenesis Index

**Figure 2:**
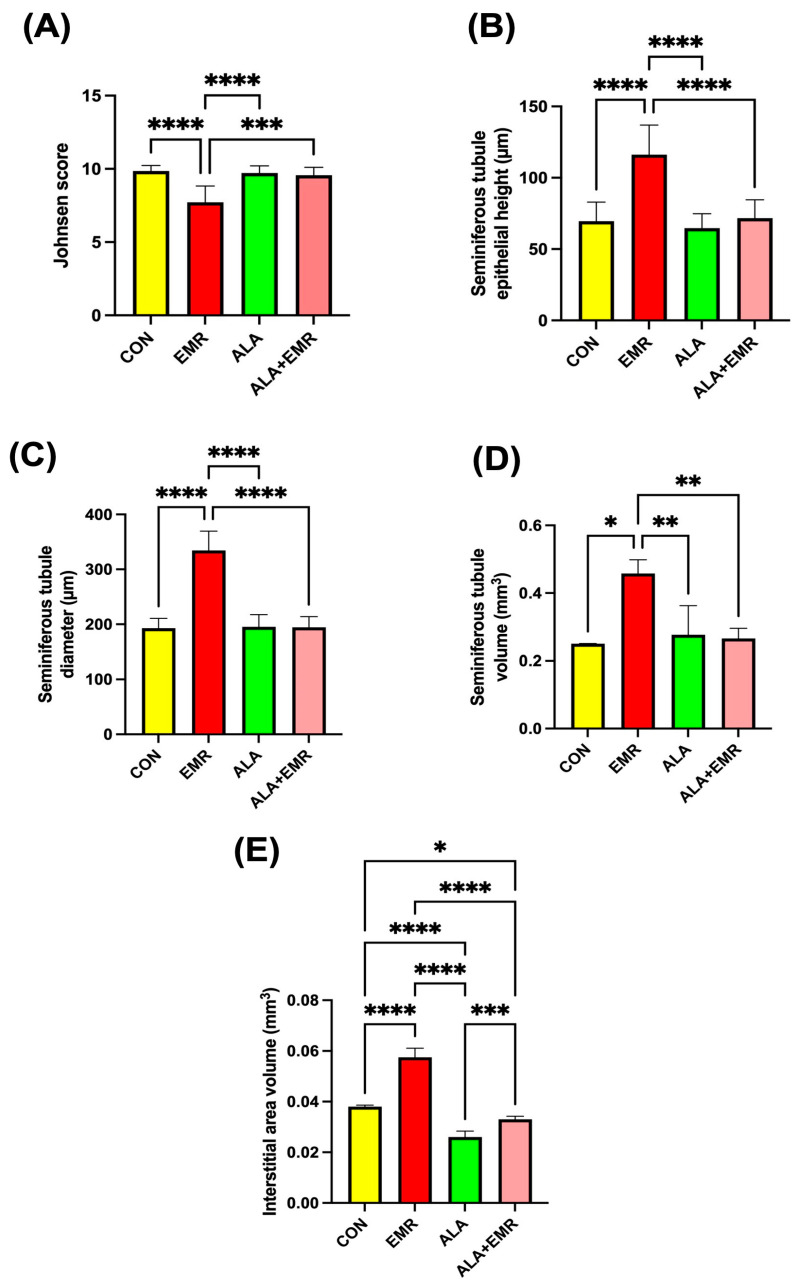
Effects of 2.45 GHz electromagnetic radiation (EMR) and alpha-lipoic acid (ALA) on spermatogenic activity and morphometric parameters in testicular tissue. (**A**) Johnsen score indicating spermatogenic efficiency. (**B**) Seminiferous tubule epithelial height. (**C**) Seminiferous tubule diameter. (**D**) Seminiferous tubule volume. (**E**) Interstitial area volume. Data are presented as mean ± SEM (*n* = 7). Statistical significance was assessed using ANOVA with Tukey’s post hoc test or unpaired *t*-test where appropriate; * *p* < 0.05, ** *p* < 0.01, *** *p* < 0.001, **** *p* < 0.0001.

Spermatogenic activity was assessed in all groups using the Johnsen scoring system. Analyses based on seminiferous tubules randomly selected from each animal revealed statistically significant differences in Johnsen scores. Johnsen scoring revealed a substantial reduction in spermatogenic activity in the EMR group compared with the CON and ALA groups (Figure 2A, **** *p* < 0.0001), indicating a significant disruption of germ cell maturation (Figure 2A). However, ALA co-treatment (ALA+EMR) markedly improved Johnsen scores relative to EMR alone (Figure 2A, *** *p* < 0.001), suggesting partial restoration of spermatogenesis.

### 3.5. Effects of 2.45 GHz EMR on Histomorphometric and Stereological Alterations in Testicular Tissue

Stereological analysis based on the Cavalieri principle has revealed that EMR exposure significantly alters testicular histomorphometry. Compared with the CON and other groups, the EMR group showed notable increases in seminiferous tubule diameter, epithelial height, and total tubule volume (Figure 2). Morphometric analysis demonstrated that seminiferous tubule epithelial height (Figure 2B), tubule diameter (Figure 2C), and tubule volume (Figure 2D) were all significantly increased in the EMR group (* *p* < 0.05 to **** *p* < 0.0001), indicative of tubular hypertrophy or oedema secondary to radiation-induced degeneration. These pathological enlargements were markedly attenuated in the ALA+EMR group (*p* < 0.01), approaching control values. Furthermore, interstitial area volume (Figure 2E) was expanded significantly in EMR-exposed rats (**** *p* < 0.0001), reflecting stromal inflammation and extracellular matrix remodelling. ALA treatment was effective in reducing tissue remodelling, as evidenced by a substantial reduction in interstitial expansion compared with the EMR group (*** *p* < 0.001).

### 3.6. Effects of 2.45 GHz EMR on Hormonal Receptors, Cell Proliferation, Inflammation, and Barrier Integrity Markers

**Figure 3:**
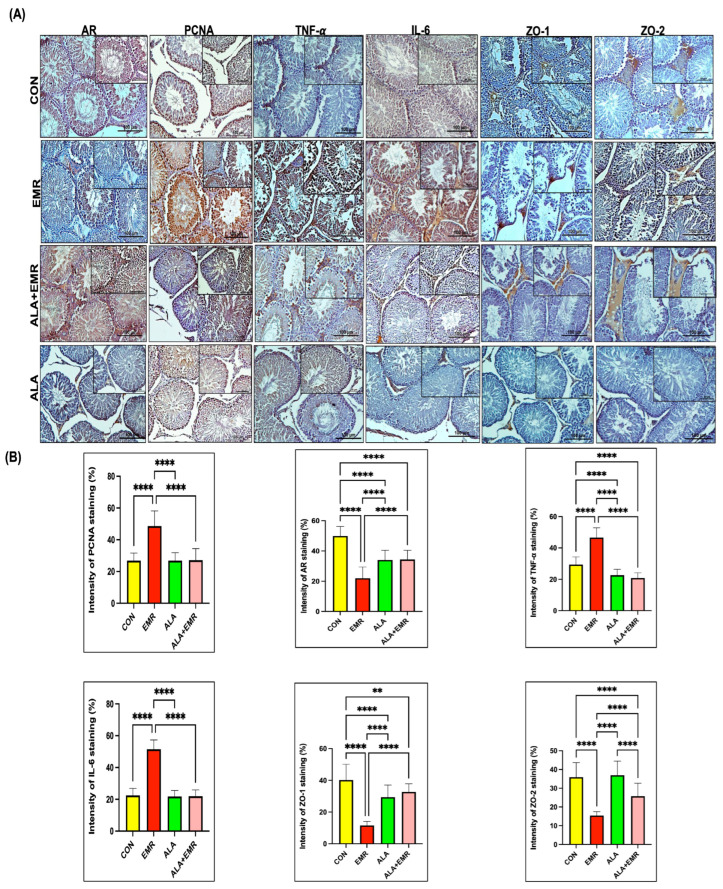
Immunohistochemical (IHC) evaluation of testicular expression of AR, PCNA, TNF-α, IL-6, ZO-1, and ZO-2 following 2.45 GHz EMR exposure and ALA treatment. (**A**) Representative IHC staining of androgen receptor (AR), proliferating cell nuclear antigen (PCNA), tumour necrosis factor-alpha (TNF-α), interleukin-6 (IL-6), and tight junction proteins (ZO-1 and ZO-2) across experimental groups (CON, EMR, ALA, and ALA+EMR) (IHC staining, Scale bar: 100 μm and 50 μm, Magnification: ×20 and ×40). (**B**) Quantitative assessment of IHC staining intensity confirms significant upregulation of inflammatory markers (TNF-α, IL-6) and suppression of AR, PCNA, ZO-1, and ZO-2 in the EMR group compared to all other groups. ALA treatment significantly reversed EMR-induced dysregulation. Data represent mean ± SEM (*n* = 7). IHC staining intensities were compared using an unpaired Student’s *t*-test; significance thresholds are reported as ** *p* ≤ 0.01, and **** *p* ≤ 0.0001.

The expression levels of AR, PCNA, TNF-α, IL-6, ZO-1, and ZO-2 were assessed in the testicular tissue following 2.45 GHz EMR exposure (Figure 3A,B). The immunohistochemical staining intensities of AR, PCNA, TNF-α, IL-6, ZO-1, and ZO-2 were compared across all groups (Figure 3). AR positivity was prominent in the seminiferous tubules, primarily in germinal epithelial cells, particularly in primary spermatogonia, and was also detected in Sertoli cells, Leydig cells, vascular endothelial cells, and myoepithelial cells. AR staining intensity was highest in the CON group, decreasing in the ALA and ALA+EMR groups; the lowest intensity was observed only in the EMR group (Figure 3B, **** *p* < 0.0001). PCNA-positive cells, reflecting proliferative activity, were observed in all groups. At the same time, a marked increase in PCNA staining intensity was detected in the spermatogenic series in the EMA group. In the ALA+EMR group, this intensity was significantly reduced (Figure 3B, **** *p* < 0.0001). IL-6 and TNF-α expression, markers of the inflammatory response, were significantly increased in the EMR group compared with the CON group (Figure 3B, **** *p* < 0.0001). This increase was significantly suppressed in the ALA-treated group (Figure 3B, **** *p* < 0.0001). Immunostaining of ZO-1 and ZO-2, which play a role in the structural integrity of the blood-testis barrier, was regularly observed in the basolateral regions of Sertoli cells in the CON and ALA groups. Conversely, the EMR group exhibited both reduced labelling intensity and significant abnormalities in the distribution pattern (Figure 3B; ** *p* ≤ 0.001). The ALA+EMR group showed a pattern similar to that of the CON group. Overall, these results confirm that ALA efficiently reverses 2.45 GHz EMR-mediated disruptions in androgen receptor signalling, inflammation, and epithelial barrier stability.

### 3.7. Effects of 2.45 GHz EMR on Serum Testosterone and Oxidative Stress Markers

**Figure 4:**
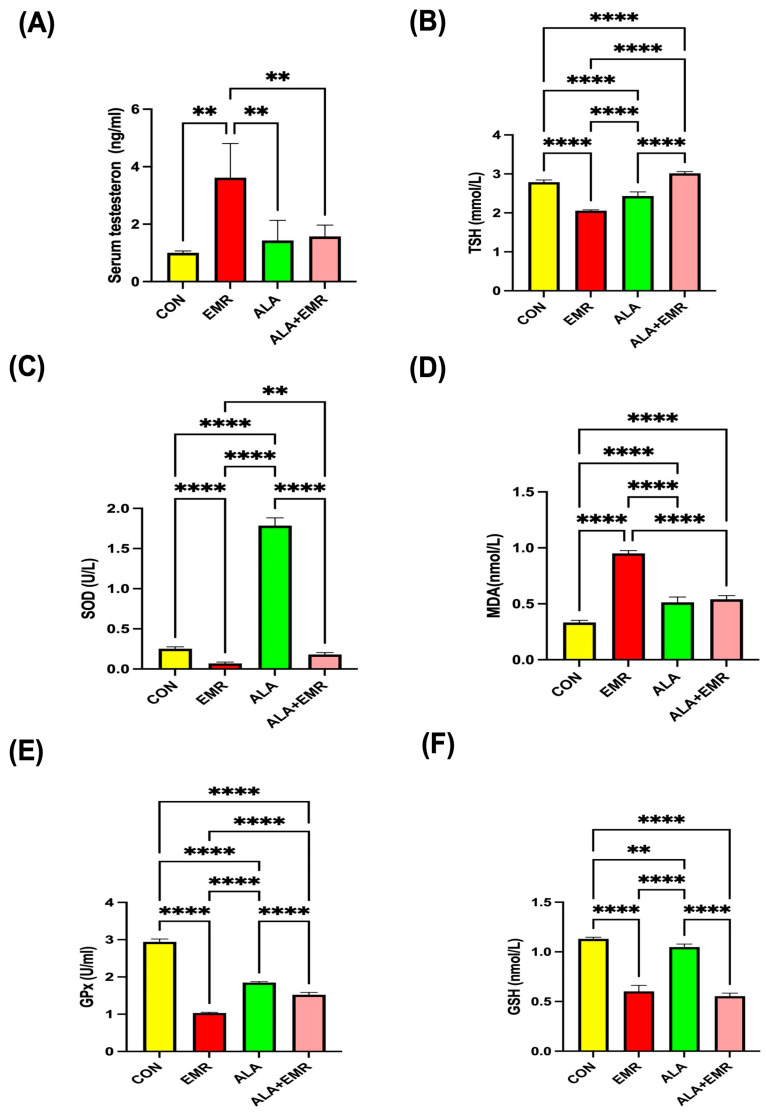
Effects of 2.45 GHz electromagnetic radiation (EMR) and alpha-lipoic acid (ALA) on serum testosterone and oxidative stress parameters in all groups. (**A**) Serum testosterone levels. (**B**) Thyroid-stimulating hormone (TSH). (**C**) Superoxide dismutase (SOD) activity. (**D**) Malondialdehyde (MDA), indicating lipid peroxidation. (**E**) Glutathione peroxidase (GPx). (**F**) Glutathione (GSH) concentrations. EMR exposure significantly increased testosterone and MDA levels while markedly suppressing antioxidant markers (SOD, GPx, and GSH). Co-administration of ALA (ALA+EMR) reversed these effects, restoring hormonal and redox balance toward control values. Data are presented as mean ± SEM (*n* = 7). Statistical significance was assessed using ANOVA with Tukey’s post hoc test or unpaired *t*-test where appropriate; ** *p* < 0.01, and **** *p* < 0.0001.

Serum testosterone levels were significantly elevated in the EMR group compared with the CON group (** *p* < 0.01), indicating a possible compensatory endocrine response to radiation-induced stress (Figure 4A). The ALA+EMR group effectively normalised testosterone levels, showing a significant reduction compared with the EMR group (** *p* < 0.01). Simultaneously, ALA treatment without EMR showed no difference from the control group.

Thyroid-stimulating hormone (TSH) concentrations were markedly reduced in the EMR group relative to all other groups (Figure 4B, **** *p* < 0.0001). Conversely, ALA therapy reinstated TSH levels to baseline, indicating a stabilising influence on systemic metabolic control.

Oxidative stress profiling revealed a profound depletion of antioxidant enzymes following exposure to 2.45 GHz EMR. Serum SOD activity exhibited the lowest values in the EMR group (Figure 4C, ** *p* < 0.01 vs. all groups), while the ALA+EMR group showed a significant recovery (** *p* < 0.01) (Figure 4C). In contrast, the ALA-only group displayed the highest SOD activity, exceeding both CON and EMR-exposed rats (Figure 4C, **** *p* < 0.0001), reflecting the strong antioxidative capacity of ALA. Serum MDA, a marker of lipid peroxidation, was dramatically elevated in the EMR group (Figure 4D, **** *p* < 0.0001 vs. all other groups), indicating intense oxidative damage (Figure 4D). ALA supplementation significantly reduced MDA levels compared with the EMR group (Figure 4D, **** *p* < 0.0001), restoring them to near-control values. Similarly, GPx and GSH levels were significantly suppressed in the EMR-exposed rats (Figure 4E,F, **** *p* < 0.0001). In contrast, both parameters were substantially improved in the ALA+EMR group (**** *p* < 0.0001) (Figure 4E,F). Taken together, these findings demonstrate that exposure to 2.45 GHz EMR induces a systemic oxidative imbalance and endocrine disruption. In contrast, ALA effectively counteracts these alterations by restoring antioxidant defences and normalising hormonal profiles.

**Figure 5:**
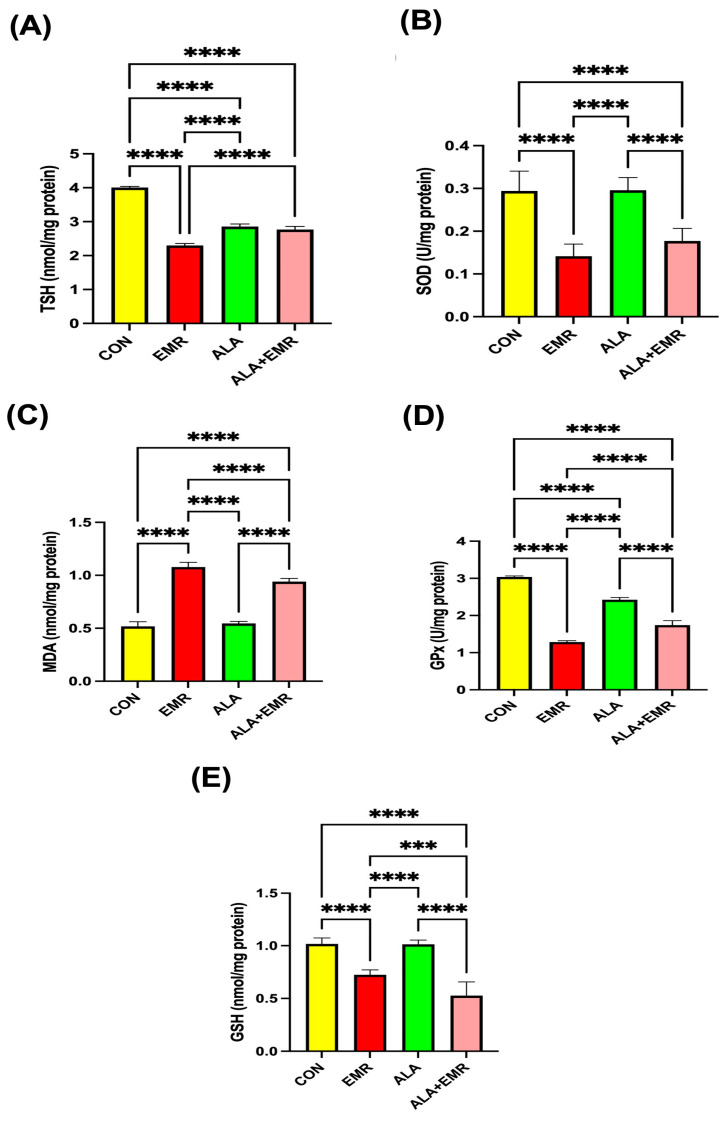
Effects of 2.45 GHz electromagnetic radiation (EMR) and alpha-lipoic acid (ALA) on oxidative stress and antioxidant parameters in testicular tissue of all groups. (**A**) Thyroid-stimulating hormone (TSH). (**B**) Superoxide dismutase (SOD) activity. (**C**) Malondialdehyde (MDA), an indicator of lipid peroxidation. (**D**) Glutathione peroxidase (GPx). (**E**) Glutathione (GSH) levels. EMR exposure significantly decreased antioxidant enzyme activities (SOD, GPx, GSH) and TSH levels while markedly increasing MDA concentrations, indicating severe redox imbalance. Co-treatment with ALA (ALA+EMR) partially or fully restored antioxidant status and reduced lipid peroxidation relative to EMR alone. Data are presented as mean ± SEM (*n* = 7); Statistical significance was assessed using ANOVA with Tukey’s post hoc test or unpaired *t*-test where appropriate; *** *p* < 0.001, and **** *p* < 0.0001.

Testicular oxidative stress markers exhibited a pattern consistent with serum findings, indicating a substantial local impact of EMR exposure on redox homeostasis. TSH levels were significantly reduced in the EMR group compared with all other groups (**** *p* < 0.0001). In contrast, ALA administration partially restored TSH concentrations toward control values (Figure 5A). SOD activity in testicular tissue was markedly suppressed by EMR (**** *p* < 0.0001), showing the lowest antioxidant enzyme capacity among all groups (Figure 5B). Co-treatment with ALA significantly improved SOD activity (**** *p* < 0.0001 vs. EMR). Lipid peroxidation levels (MDA) were highest in the EMR group (**** *p* < 0.0001), confirming intense oxidative damage within testicular tissue (Figure 5C). ALA supplementation significantly reduced MDA concentrations, demonstrating a potent protective effect against EMR-induced lipid oxidation. Similarly, GPx and GSH levels were profoundly diminished in the EMR group (**** *p* < 0.0001), reflecting impaired antioxidant defence (Figure 5D,E). ALA co-administration effectively restored GPx and GSH levels. However, the values remained slightly lower than those of the CON group. No significant differences were detected between the ALA-only and CON groups for any antioxidant parameter. Taken together, these results indicate that 2.45 GHz EMR induces pronounced oxidative damage in testicular tissue by suppressing endogenous antioxidant defences and elevating lipid peroxidation. In contrast, ALA confers substantial protection by restoring redox balance. Table 1 summarises the biochemical results across the experimental groups.

## 4. Discussion

Electromagnetic radiation (EMR) has become one of the most prevalent environmental stressors of the modern era due to the widespread use of wireless communications, medical devices, industrial systems, and defence technologies. While international exposure guidelines primarily focus on thermal thresholds, increasing experimental and epidemiological findings suggest that long-term, low-intensity EMR can produce non-thermal biological effects, including increased oxidative stress, triggered inflammatory responses, and disrupted cellular barrier integrity [34,35]. The testis is particularly susceptible to these effects due to its high metabolic activity, intense mitochondrial function, and dependence on delicate paracrine communication in the seminiferous epithelium. Consequently, both germ cells and the blood-testis barrier (BTB) are vulnerable to disruptions caused by reactive oxygen species and inflammatory mediators, rendering spermatogenesis fragile to EMR-induced irregularities [34,36]. 2.45 GHz EMR emitted from Wi-Fi routers, Bluetooth devices, and microwave systems commonly used in daily life has been reported to cause lipid peroxidation, germ cell loss, Sertoli cell dysfunction, and decreased sperm quality in rodent models; these findings point to a possible mechanistic link between EMR exposure and reproductive dysfunction [1,37]. Therefore, 2.45 GHz EMR, which reflects real-life exposure and reliably demonstrates biological changes in preclinical models, was preferred for this study [38,39,40]. Uncertainties regarding non-thermal effects, often overlooked in regulatory assessments, underscore the importance of mechanistic investigations in testicular biology. In this context, our study aimed to characterise the impact of 2.45 GHz EMR on testicular redox balance, barrier protein expression, and spermatogenic architecture using a multi-parameter biomarker approach, and to evaluate the potential protective role of ALA, a clinically recognised endogenous antioxidant, against EMR-induced impairments.

Recent evidence suggests that EMR influences not only localised tissues but also systemic homeostatic pathways [41,42,43]. Consistent with this, 2.45 GHz EMR exposure in our study induced both metabolic and reproductive disturbances. The increase in water intake may reflect osmoregulatory stress mediated by hypothalamic–hypophyseal activation, as previously proposed [44,45]. However, contrasting reports of hypodipsic responses under different EMR conditions highlight that hydration behaviour is highly dependent on exposure duration, field intensity, and thermal versus non-thermal components [41,43,46]. Fluctuations in body weight further support the presence of a systemic stress response. The transient weight gain observed on day 15, followed by stabilisation, resembles early compensatory hypermetabolism described in short-term EMR exposures, whereas long-term models often report progressive catabolic outcomes [47,48]. Such divergence suggests that EMR-induced metabolic alterations may follow a dynamic rather than a linear trajectory. Regarding reproductive outcomes, the increase in testicular mass may appear to reflect compensatory hypertrophy; however, its coexistence with interstitial oedema, collagen deposition, and disrupted spermatogenesis indicates a pathological rather than adaptive process [49,50]. Overall, these findings demonstrate that 2.45 GHz EMR perturbs systemic homeostasis and impairs testicular microarchitecture.

Increasing numbers of experimental studies have demonstrated that 2.45 GHz EMR disrupts male reproductive homeostasis by inducing oxidative and inflammatory injury in the testes. Consistent with previous histopathological reports, EMR exposure has been associated with disorganisation of the spermatogenic epithelium, degeneration of seminiferous tubules, atrophic changes, and marked reductions in Johnsen’s score, all indicative of impaired germ cell maturation and decreased sperm output [1,2,4,7,40,47,49]. Structural alterations also extend to the interstitial compartment, where extracellular matrix remodelling—including collagen accumulation, tubular dilation, and epithelial thickening—has been documented. Stereological findings further support disproportionate increases in seminiferous and interstitial volumes, reflecting inflammatory oedema and fibrotic remodelling rather than functional hypertrophy. Our results are in strong agreement with this pathological profile. In the EMR group, decreased Johnsen’s scores, increased epithelial height and tubular volume, interstitial expansion, and enhanced collagen deposition collectively confirm EMR-induced impairment of spermatogenesis and stromal fibrosis. The degenerative tubular patterns observed in our study are consistent with prior evidence of elevated germ cell apoptosis under similar exposure conditions. Significantly, ALA co-treatment markedly ameliorated these EMR-induced alterations, restoring seminiferous architecture, improving germ cell progression, and attenuating interstitial collagen accumulation. While melatonin and gallic acid have been shown to confer partial protection primarily through antioxidant pathways [7,8], ALA appears to exert a broader, multi-target effect. Beyond its well-established capacity to reinforce redox balance, ALA stabilises epithelial and interstitial structures, preserves microenvironmental integrity, and suppresses fibrotic remodelling. These findings position ALA as a potent multifunctional agent capable of simultaneously mitigating oxidative stress, protecting germinal epithelium, and restraining pathological tissue remodelling.

EMR exposure has been shown to alter the expression of key regulators of testicular function, particularly PCNA and AR, with EMR-driven PCNA overexpression and AR downregulation associated with impaired reproductive capacity and potential carcinogenic risk [51,52,53]. Previous experimental studies also demonstrate that 2.45 GHz EMR disrupts critical immunohistochemical markers in testicular tissue, indicating that its detrimental effects extend beyond oxidative stress to include inflammatory activation and barrier dysfunction [54,55,56]. Consistent with this evidence, our findings confirm that EMR induces a multifactorial toxic response characterised by increased TNF-α and IL-6 expression, reduced AR levels, and downregulation of tight-junction proteins such as ZO-1 and ZO-2 [57,58]. These alterations collectively reflect compromised BTB integrity, heightened inflammatory signalling, and impaired endocrine responsiveness. Although PCNA expression showed some variability, the reduction in Johnsen scores and histological deterioration suggests inadequate compensatory proliferation. Significantly, ALA effectively counteracted these EMR-induced disturbances. Beyond its established antioxidant role, ALA suppressed inflammatory cytokine upregulation, restored AR and tight-junction protein expression, and supported epithelial and hormonal homeostasis. Compared with agents such as melatonin and gallic acid, which primarily modulate oxidative pathways [7,8], ALA exhibited a broader protective profile by simultaneously improving redox balance, epithelial stability, and androgen signalling. These integrated effects highlight ALA as a promising therapeutic candidate for mitigating EMR-induced testicular dysfunction.

Our current findings support the growing evidence that exposure to 2.45 GHz EMR disrupts testicular homeostasis through interconnected endocrine and redox mechanisms. Much of the literature has reported decreases in serum testosterone following EMR exposure, associated with Leydig cell damage and decreased 3β-HSD activity [59,60]. In contrast, the early-phase increase in testosterone observed in our study is consistent with a temporary compensatory endocrine response to EMR-induced oxidative stress. Such short-term increases reflect adaptive mechanisms aimed at maintaining steroidogenic signalling but are often overlooked in shorter-term or lower-intensity exposures. The early increase in serum testosterone at 2.45 GHz EMR appears consistent with compensatory HPG axis activation triggered by stress responses specific to the acute phase of exposure. It has previously been suggested that acute oxidative stress may cause a short-term rise in LH by stimulating hypothalamic GnRH release, which, in turn, could lead to a transient increase in testosterone in Leydig cells [61]. Similarly, short-term increases in testosterone associated with LH elevation have been reported in acute heat or oxidative stress models [62]. This mechanism may explain the discrepancy between studies reporting testosterone suppression, particularly with prolonged exposure [61,63], and studies observing testosterone increases with acute exposure. In our study, ALA’s suppression of EMR-induced testosterone elevation is consistent with ALA reducing oxidative stress and thereby eliminating compensatory stimulation of the HPG axis. Various experimental models have demonstrated that ALA both enhances antioxidant defence and suppresses the stress-induced upregulation of GnRH/LH signalling [13,15,64]. Furthermore, it has been reported that ALA protects Leydig cells from oxidative damage, normalises the overactivation or inhibition of steroidogenesis enzymes, and stabilises hormonal fluctuations [64,65,66]. Therefore, the normalisation of testosterone levels in the ALA + EMR group supports the protective role of ALA in suppressing excessive HPG axis activation by restoring redox balance.

In our study, the reduction in serum TSH after EMR exposure suggests a possible involvement of the hypothalamic–pituitary–thyroid (HPT) axis, as TSH is a central regulator of thyroid hormone synthesis. Although EMR research has predominantly focused on reproductive endpoints, emerging evidence indicates that EMR may also affect thyroid-regulating pathways through oxidative stress or autonomic imbalance (e.g., changes in TSH or thyroid hormone profiles) [4,67,68,69]. Therefore, the decrease in TSH observed in our model may reflect an early stress-related suppression of the HPT axis rather than a primary reproductive effect. Given the well-defined roles of thyroid hormones in Sertoli cell maturation, energy metabolism, and steroidogenesis [67], these alterations in the HPT axis may represent a critical pathway in reproductive EMR toxicology that has been insufficiently investigated and could contribute to gonadal endocrine imbalance. Indeed, the literature on testosterone alterations is heterogeneous [70,71,72], with results significantly dependent on factors such as exposure duration, SAR value, tissue thermal load, and the organism’s oxidative stress tolerance.

Studies reporting decreases in testosterone following long-term or high-intensity exposure to 2.45 GHz EMR [40,70,72,73] are explained by Leydig cell dysfunction and chronic suppression of the hypothalamic–pituitary–gonadal (HPG) axis. Conversely, studies have also identified transient increases in testosterone levels in shorter-term or lower SAR protocols; these increases are interpreted as adaptive or compensatory activation of the HPG axis triggered by acute radiation-induced oxidative stress [71,74]. In this context, the increase in testosterone observed in the EMR group in our study should be interpreted not as a sustained stimulatory effect but rather as an early-stage endocrine adaptation aimed at balancing oxidative load. This interpretation is also consistent with models suggesting that acute EMR exposure may initially activate feedback mechanisms, but that this activation is suppressed under chronic exposure conditions.

Consistent with previous studies demonstrating increased lipid peroxidation and weakened antioxidant defences under EMR exposure [7,75,76], oxidative stress emerged as a central driver of injury in our model. Reductions in SOD, GSH, and GPx, along with elevated MDA, revealed a pronounced redox imbalance—more severe in testicular tissue than in circulation—highlighting the heightened sensitivity of the local gonadal microenvironment and the limitations of relying solely on systemic biomarkers. Therapeutic ALA administration effectively restored antioxidant capacity, reduced MDA accumulation, and normalised both testosterone and TSH levels. Compared with protective agents such as melatonin or gallic acid, which primarily target oxidative pathways [7,8], ALA exhibited broader efficacy by simultaneously modulating redox state, endocrine outputs, and metabolic equilibrium. This multimodal action underscores ALA as a promising candidate for mitigating EMR-induced testicular dysfunction. Variability in hormonal outcomes reported across studies likely reflects differences in exposure duration, intensity, animal age, and sampling time points. Our data emphasise the need for dose–response and time-course studies to clarify the biphasic endocrine patterns associated with EMR exposure, as well as the importance of tissue-specific biochemical and histological evaluations. Overall, our findings support the view that 2.45 GHz EMR induces testicular injury through intersecting oxidative, endocrine, and metabolic pathways [7,75,76], while demonstrating that ALA not only restores redox balance but also corrects EMR-related hormonal disturbances, including early compensatory testosterone surges and HPT axis suppression. These results broaden current perspectives on EMR toxicity and highlight antioxidant–endocrine–targeted strategies as a rational therapeutic avenue.

The translational relevance of these findings holds substantial implications for human reproductive health. The observed alterations in inflammatory signalling, oxidative stress dynamics, and tissue-specific structural responses suggest mechanistic pathways that may similarly operate in human reproductive organs exposed to comparable environmental or pathological stressors. Given that dysregulated inflammatory and oxidative processes are strongly associated with impaired gametogenesis, disrupted steroidogenesis, and reduced fertility potential in humans, the present results provide a biological framework that can inform future clinical investigations. Importantly, the molecular signatures identified in this experimental model may serve as early biomarkers of reproductive dysfunction and guide the development of targeted therapeutic strategies to preserve reproductive capacity in at-risk populations.

This study has several limitations that should be acknowledged. First, our experimental design focused on acute changes following 30 days of exposure to 2.45 GHz EMR. In contrast, many reproductive toxicology studies have employed longer or repeated exposure protocols and have demonstrated more pronounced or persistent impairments in sperm quality and fertility-related outcomes [1,2,3,4]. Consequently, it remains to be determined whether the protective effects of ALA observed here are sustained under chronic EMR conditions or entirely prevent long-term subfertility. Second, we administered a single fixed dose of ALA; however, preclinical data indicate that the antioxidant and cytoprotective actions of ALA on male reproductive endpoints may be dose- and model-dependent, and optimal dosing regimens (including potential benefits or ceiling effects at higher doses) remain to be defined [5,6,7,8]. Third, we did not directly assess functional fertility parameters such as mating success, in vivo fertilising capacity, or detailed sperm functional assays (e.g., sperm–oocyte interaction), which are increasingly emphasised as critical readouts in EMR-related male reproductive toxicology [4,9,10]. These gaps should be addressed in future studies to evaluate whether ALA can reliably mitigate EMR-induced reproductive dysfunction more comprehensively and to support eventual clinical translation. 

## 5. Conclusions

Overall, our findings demonstrate that ALA provides multi-dimensional protection against 2.45 GHz EMR-induced reproductive toxicity by modulating oxidative stress, attenuating inflammatory signalling, preserving testicular architecture, and stabilising key endocrine pathways. The present controlled rat model indicates that EMR disrupts testicular homeostasis through interconnected oxidative, inflammatory, and hormonal mechanisms, ultimately impairing spermatogenic activity and compromising blood–testis barrier integrity. Co-administration of ALA restored antioxidant defences, reduced pro-inflammatory responses, maintained tight-junction structure, and normalised endocrine parameters, suggesting actions that extend beyond simple radical scavenging. While these results position ALA as a promising preclinical candidate for mitigating EMR-associated gonadal dysfunction, they should be interpreted with the limitations of the present model in mind. Long-term fertility assessments, dose–response optimisation, mechanistic pathway analyses, and extended exposure paradigms will be required to fully determine its translational relevance.

## Figures and Tables

**Table 1:** Summary of key histopathological and biochemical findings across experimental groups.

Parameter	CON	EMR (2.45 GHz)	ALA	ALA + EMR
General Testicular Histology	Normal architecture; intact tunica albuginea, seminiferous tubules, germinal epithelium	Severe structural damage; epithelial detachment, reduced epithelial thickness, germ cell loss	Normal histomorphology; no degeneration	Structural damage markedly reduced; near-normal architecture
Basal Lamina Integrity	Regular, continuous	Separated, irregular	Normal	Partially restored; less disruption
Germ Cell Loss	None	Marked loss	None	Mild–moderate loss; significantly reduced vs. EMR
Interstitial Oedema and Congestion	Absent	Severe	Absent	Mild; reduced vs. EMR
Vacuolization in Tubular Epithelium	None	Pronounced	None	Reduced intensity
Interstitial Connective Tissue	Normal	Decreased	Normal	Partially restored
Collagen Accumulation (MT staining)	Minimal, physiological distribution	Strongly increased; dense bundles around tubules and vasculature	Minimal	Significantly reduced compared to EMR; near-normal fine fibres
Serum Testosterone	Normal baseline	Increased (** *p* < 0.01)	Normal	Normalised; reduced vs. EMR (** *p* < 0.01)
Serum TSH	Normal	Strongly decreased (**** *p* < 0.0001)	Normal	Restored toward control
Serum SOD	Normal	Lowest level (** *p* < 0.01)	Highest level (**** *p* < 0.0001)	Significant recovery
Serum MDA	Low baseline	Highest level (**** *p* < 0.0001)	Low	Markedly reduced; near control
Serum GPx	Normal	Suppressed (**** *p* < 0.0001)	Normal	Restored; slightly lower than control
Serum GSH	Normal	Suppressed (**** *p* < 0.0001)	Normal	Significant recovery
Testicular SOD	Normal	Strongly suppressed (**** *p* < 0.0001)	Normal	Partially restored
Testicular MDA	Low	Highest (**** *p* < 0.0001)	Low	Reduced vs. EMR; protective effect
Testicular GPx	Normal	Lowest (**** *p* < 0.0001)	Normal	Improved but not fully normalised
Testicular GSH	Normal	Lowest (**** *p* < 0.0001)	Normal	Restored; slight deficit remains

## Data Availability

The original contributions presented in this study are included in the article/Supplementary Material. Further inquiries can be directed to the corresponding author.

## Supplementary Materials

The following supporting information can be downloaded at: https://www.mdpi.com/article/10.3390/biomedicines13123089/s1. Figure S1 Evaluation of physiological parameters following 2.45 GHz EMR exposure and ALA treatment. (A) Percentage change in body weight measured on the 15th and 30th day of the experimental period. EMR-exposed rats showed a significant increase in body weight gain on day 15 compared with the CON and ALA groups, whereas this change was not significant on day 30. Co-administration of ALA (ALA+EMR) attenuated EMR-induced body weight elevation at both time points. (B) Average daily water consumption. EMR exposure markedly increased fluid intake, suggesting a stress-related compensatory metabolic response. ALA treatment significantly reduced this elevation in the ALA+EMR group, restoring values to baseline levels. (C) Final testis mass at sacrifice. EMR-exposed animals displayed a mild but significant increase in testis weight compared to controls, whereas ALA co-treatment normalised testicular mass toward physiological levels. Data are presented as mean ± SEM. * *p* < 0.05, ** *p* < 0.01, *** *p* < 0.001, **** *p* < 0.0001, ns: not significant.

## References

[1] Assefa E.M., Abdu S.M. (2025). Histopathologic effects of mobile phone radiation exposure on the testes and sperm parameters: A systematic literature review of animal studies. Front. Reprod. Health.

[2] Jamaludin N., Ibrahim S.F., Jaffar F.H.F., Zulkefli A.F., Osman K. (2025). The Influence of 2.45 GHz Wi-Fi Exposure Duration on Sperm Quality and Testicular Histopathology: An Exploration of Peroxidative Injury. Antioxidants.

[3] Kaltsas A., Markou E., Kyrgiafini M.-A., Zikopoulos A., Symeonidis E.N., Dimitriadis F., Zachariou A., Sofikitis N., Chrisofos M. (2025). Oxidative-Stress-Mediated Epigenetic Dysregulation in Spermatogenesis: Implications for Male Infertility and Offspring Health. Genes.

[4] Pawlak K., Bojarski B., Jagusiak W., Wojnar T., Nieckarz Z., Arent Z., Ludwiczak M., Lasko M. (2025). An 1800 MHz electromagnetic field affects hormone levels, sperm quality, and behavior in laboratory rats (*Rattus norvegicus*). Appl. Sci..

[5] Potiris A., Moustakli E., Trismpioti E., Drakaki E., Mavrogianni D., Matsas A., Zikopoulos A., Sfakianakis A., Tsakiridis I., Dagklis T. (2025). From Inflammation to Infertility: How Oxidative Stress and Infections Disrupt Male Reproductive Health. Metabolites.

[6] Safaeinezhad A., Mousapour E., Baba Ahmadi A.K., Ebrahimi S., Rahimi K., Rezaie A., Dehvari M., Givi M.E., Sasani A. (2025). Effects of coenzyme Q10 on sperm parameters and pathological changes induced by Wi-Fi waves in the testicular tissue of rats. Ann. Med. Surg..

[7] Saygin M., Asci H., Ozmen O., Cankara F.N., Dincoglu D., Ilhan I. (2016). Impact of 2.45 GHz microwave radiation on the testicular inflammatory pathway biomarkers in young rats: The role of gallic acid. Environ. Toxicol..

[8] Oksay T., Naziroğlu M., Doğan S., Güzel A., Gümral N., Koşar P.A. (2014). Protective effects of melatonin against oxidative injury in rat testis induced by wireless (2.45 GHz) devices. Andrologia.

[9] Saygin M., Ozmen O., Erol O., Ellidag H.Y., Ilhan I., Aslankoc R. (2018). The impact of electromagnetic radiation (2.45 GHz, Wi-Fi) on the female reproductive system: The role of vitamin C. Toxicol. Ind. Health.

[10] Pires I.Z., Gobbo M.O.d.S., Sudo R.Y.U., Milbradt T.L., Marquardt Filho N., Carvalhal G.F., Ros C.T.D. (2025). Efficacy of Alpha Lipoic Acid Supplementation in Sperm Parameters: A Systematic Review and Meta-Analysis of Randomized Trials. Int. Braz. J. Urol..

[11] Udaipurwala A., Pimple P., Sawarkar S. (2025). Exploring the Pharmacotherapeutic Potential of Natural Compound Alpha Lipoic Acid. Nat. Prod. J..

[12] Selvakumar E., Prahalathan C., Mythili Y., Varalakshmi P. (2004). Protective effect of dl-α-lipoic acid in cyclophosphamide induced oxidative injury in rat testis. Reprod. Toxicol..

[13] Takeda T., Matsuo Y., Nishida K., Fujiki A., Hattori Y., Koga T., Ishii Y., Yamada H. (2017). α-Lipoic acid potentially targets AMP-activated protein kinase and energy production in the fetal brain to ameliorate dioxin-produced attenuation in fetal steroidogenesis. J. Toxicol. Sci..

[14] Pınar N., Çakırca G., Özgür T., Kaplan M. (2018). The protective effects of alpha lipoic acid on methotrexate induced testis injury in rats. Biomed. Pharmacother..

[15] Namoju R., Chilaka N.K. (2022). Maternal supplementation of α-lipoic acid attenuates prenatal cytarabine exposure-induced oxidative stress, steroidogenesis suppression and testicular damage in F1 male rat fetus. Beni Suef Univ. J. Basic Appl. Sci..

[16] Shahid A., Nasir K., Bhatia M. (2025). Therapeutic Potential of Alpha-Lipoic Acid: Unraveling Its Role in Oxidative Stress and Inflammatory Conditions. Curr. Issues Mol. Biol..

[17] Sun J., Malyar R.M., Ye N., Wang Y., Wei Q., Shi F., Li Y. (2025). Alpha-Lipoic Acid Alleviates Lead-Induced Testicular Damage in Roosters by Reducing Oxidative Stress and Modulating Key Pathways. Toxics.

[18] Wang Y., Jiang S., He Y., Pang P., Shan H. (2025). Advances in α-Lipoic Acid for Disease Prevention: Mechanisms and Therapeutic Insights. Molecules.

[19] Morsy M.A., Abdalla A.M., Mahmoud A.M., Abdelwahab S.A., Mahmoud M.E. (2012). Protective effects of curcumin, α-lipoic acid, and N-acetylcysteine against carbon tetrachloride-induced liver fibrosis in rats. J. Physiol. Biochem..

[20] Maciejczyk M., Żebrowska E., Nesterowicz M., Żendzian-Piotrowska M., Zalewska A. (2022). α-Lipoic Acid Strengthens the Antioxidant Barrier and Reduces Oxidative, Nitrosative, and Glycative Damage, as well as Inhibits Inflammation and Apoptosis in the Hypothalamus but Not in the Cerebral Cortex of Insulin-Resistant Rats. Oxid. Med. Cell. Longev..

[21] Dajnowicz-Brzezik P., Żebrowska E., Maciejczyk M., Zalewska A., Chabowski A. (2025). α-lipoic acid supplementation reduces oxidative stress and inflammation in red skeletal muscle of insulin-resistant rats. Biochem. Biophys. Res. Commun..

[22] Odacı E., Özyılmaz C. (2015). Exposure to a 900 MHz electromagnetic field for 1 hour a day over 30 days does change the histopathology and biochemistry of the rat testis. Int. J. Radiat. Biol..

[23] Özsobacı N.P., Ergün D.D., Tunçdemir M., Özçelik D. (2020). Protective effects of zinc on 2.45 GHz electromagnetic radiation-induced oxidative stress and apoptosis in HEK293 cells. Biol. Trace Elem. Res..

[24] Qin T., Liu L., Wang X., Guo L., Lin J., Du J., Xue Y., Lai P., Jing Y., Ding G. (2023). Combined effects of EMP and RF field on emotional behavior in mice. Front. Public Health.

[25] Keskin S., Acikgoz E., Ertürk F.Y., Ragbetli M.C., Ozkol H. (2023). Histopathological changes in liver and heart tissue associated with experimental ultraviolet radiation a and b exposure on wistar albino rats. Photochem. Photobiol..

[26] Johnsen S.G. (1970). Testicular biopsy score count—A method for registration of spermatogenesis in human testes: Normal values and results in 335 hypogonadal males. Hormones.

[27] Fu W., Cui J., Tang S. (2024). The relationship of testicular stiffness with Johnsen score and sperm retrieval outcome in men with non-obstructive azoospermia. Quant. Imaging Med. Surg..

[28] Teixeira T.A., Pariz J.R., Dutra R.T., Saldiva P.H., Costa E., Hallak J. (2019). Cut-off values of the Johnsen score and Copenhagen index as histopathological prognostic factors for postoperative semen quality in selected infertile patients undergoing microsurgical correction of bilateral subclinical varicocele. Transl. Androl. Urol..

[29] Colcimen N., Keskin S. (2025). Evaluation of the Protective Effects of Alpha Lipoic Acid on Bleomycin-Induced Ovarian Toxicity. J. Biochem. Mol. Toxicol..

[30] Baba B., Ceylani T., Teker H.T., Keskin S., Genc A.I., Gurbanov R., Acikgoz E. (2025). Therapeutic potential of young plasma in reversing age-related liver inflammation via modulation of NLRP3 inflammasome and necroptosis. Biogerontology.

[31] Schindelin J., Arganda-Carreras I., Frise E., Kaynig V., Longair M., Pietzsch T., Preibisch S., Rueden C., Saalfeld S., Schmid B. (2012). Fiji: An open-source platform for biological-image analysis. Nat. Methods.

[32] Gundersen H., BENDTSEN T.F., KORBO L., MARCUSSEN N., Møller A., Nielsen K., Nyengaard J., Pakkenberg B., Sørensen F.B., Vesterby A. (1988). Some new, simple and efficient stereological methods and their use in pathological research and diagnosis. APMIS.

[33] Gundersen H.J.G., Jensen E. (1987). The efficiency of systematic sampling in stereology and its prediction. J. Microsc..

[34] Parashar R., Yadav S.M., Meena P., Kumar R., Jheeta K.S., Saini P., Patel D.D. (2025). Response of Male Reproductive System against Ionizing Radiation and Available Radio-protective Agents: Cellular and Molecular Insight. Curr. Radiopharm..

[35] Li Y., Yao B., Men J., Pang Y., Gao J., Bai Y., Wang H., Zhang J., Zhao L., Xu X. (2025). Oxidative stress and energy metabolism in male reproductive damage from single and combined high-power microwave exposure at 1.5 and 4.3 GHz. Reprod. Toxicol..

[36] Motchidlover L., Sari-Minodier I., Sunyach C., Metzler-Guillemain C., Perrin J. (2025). Impact of non-ionising radiation of male fertility: A systematic review. Fr. J. Urol..

[37] Liu L., Huang B., Lu Y., Zhao Y., Tang X., Shi Y. (2024). Interactions between electromagnetic radiation and biological systems. Iscience.

[38] Bayat M., Karimi N., Karami M., Haghighi A.B., Bayat K., Akbari S., Haghani M. (2023). Chronic exposure to 2.45 GHz microwave radiation improves cognition and synaptic plasticity impairment in vascular dementia model. Int. J. Neurosci..

[39] Bektas H., Dasdag S. (2025). The effects of radiofrequency radiation on male reproductive health and potential mechanisms. Electromagn. Biol. Med..

[40] Shahin S., Mishra V., Singh S., Chaturvedi C. (2014). 2.45-GHz microwave irradiation adversely affects reproductive function in male mouse, Mus musculus by inducing oxidative and nitrosative stress. Free Radic. Res..

[41] Khater Z.Z., Ghareeb H.S., Ibraheim M.H. (2018). Ecotoxicological effects of electromagnetic radiation on the wild rat, rattus norvegicus. Egypt. Acad. J. Biol. Sci. B. Zool..

[42] Guy A.W., Chou C.-K., Johnson R.B., Kunz L. (1980). Study of effects of long-term low-level RF exposure on rats: A plan. Proc. IEEE.

[43] El-Bediwi A.B., Saad M., El-kott A.F., Eid E. (2013). Influence of electromagnetic radiation produced by mobile phone on some biophysical blood properties in rats. Cell Biochem. Biophys..

[44] Lai H., Levitt B.B. (2024). Cellular and molecular effects of non-ionizing electromagnetic fields. Rev. Environ. Health.

[45] de Kleijn S., Ferwerda G., Wiese M., Trentelman J., Cuppen J., Kozicz T., de Jager L., Hermans P.W., Verburg-van Kemenade B.M. (2016). A short-term extremely low frequency electromagnetic field exposure increases circulating leukocyte numbers and affects HPA-axis signaling in mice. Bioelectromagnetics.

[46] Türedi S., Kerimoğlu G., Mercantepe T., Odacı E. (2017). Biochemical and pathological changes in the male rat kidney and bladder following exposure to continuous 900-MHz electromagnetic field on postnatal days 22–59. Int. J. Radiat. Biol..

[47] Sani A., Labaran M., Dayyabu B. (2018). Effects of Electromagnetic Radiation of Mobile Phones on Hematological and Biochemical Parameters in Male Albino Rats. Eur. J. Exp. Biol..

[48] Maalouf J., Pelletier A., Corona A., Gay-Quéheillard J., Bach V., de Seze R., Selmaoui B. (2023). Dose- and Time-Dependent Effects of Radiofrequency Electromagnetic Field on Adipose Tissue: Implications of Thermoregulation and Mitochondrial Signaling. Int. J. Mol. Sci..

[49] Chauhan P., Verma H., Sisodia R., Kesari K. (2016). Microwave radiation (2.45 GHz)-induced oxidative stress: Whole-body exposure effect on histopathology of Wistar rats. Electromagn. Biol. Med..

[50] Almášiová V., Holovska K., Šimaiová V., Beňová K., Raček A., Eniko R., Martoncíková M., Mihalik J., Horváthová F., Tarabová L. (2017). The thermal effect of 2.45 GHz microwave radiation on rat testes. Acta Vet. Brno.

[51] Zhao W.P., Wang H.W., Liu J., Tan P.P., Luo X.L., Zhu S.Q., Chen X.L., Zhou B.H. (2018). Positive PCNA and Ki-67 Expression in the Testis Correlates with Spermatogenesis Dysfunction in Fluoride-Treated Rats. Biol. Trace Elem. Res..

[52] Lu S., Dong Z. (2021). Proliferating cell nuclear antigen directly interacts with androgen receptor and enhances androgen receptor-mediated signaling. Int. J. Oncol..

[53] Sepehrimanesh M., Kazemipour N., Saeb M., Nazifi S., Davis D. (2017). Proteomic analysis of continuous 900-MHz radiofrequency electromagnetic field exposure in testicular tissue: A rat model of human cell phone exposure. Environ. Sci. Pollut. Res..

[54] Cheng Q., Yu G., Wang G., Bai Z.-M. (2021). Exposure of the scrotum to 4G cellphone radiofrequency electromagnetic radiation affects the spermatogenesis and blood-testis barrier of adult male rats. Zhonghua Nan Ke Xue Natl. J. Androl..

[55] Aslan E., Er H., Ozgurses M., Taşdemir M., Ozen S., Eslamkhah S., Gungor N. (2024). Impact of Electromagnetic Field Radiation on Blood-Testis Barrier Regulatory Protein Levels in Rat Testes. Acta Sci. Med. Sci..

[56] Žura Žaja I., Martinec P., Butković I., Vilić M., Milinković-Tur S., Vince S., Sluganović A., Samardžija M., Pejakovic Hlede J., Folnozic I. (2023). Effects of radiofrequency electromagnetic radiation on male fertility. Vet. Stanica.

[57] Shen Y., You Y., Zhu K., Fang C., Yu X., Chang D. (2022). Bibliometric and visual analysis of blood-testis barrier research. Front. Pharmacol..

[58] Zhang H., Yin Y., Wang G., Liu Z., Liu L., Sun F. (2014). Interleukin-6 disrupts blood-testis barrier through inhibiting protein degradation or activating phosphorylated ERK in Sertoli cells. Sci. Rep..

[59] Lin Y.-Y., Wu T., Liu J.-Y., Gao P., Li K., Guo Q.-Y., Lang H.-Y., Yuan M., Zeng L.-H., Guo G.-Z. Testosterone secretion in mouse Leydig cells decreasing induced by Radiofrequency electromagnetic radiation. Proceedings of the 2019 IEEE MTT-S International Microwave Biomedical Conference.

[60] Shokri M., Shamsaei M.E., Malekshah A.K., Amiri F.T. (2020). The protective effect of melatonin on radiofrequency electromagnetic fields of mobile phone-induced testicular damage in an experimental mouse model. Andrologia.

[61] Siegel R., Weidenfeld J., Feldman S., Conforti N., Chowers I. (1981). Neural pathways mediating basal and stress-induced secretion of luteinizing hormone, follicle-stimulating hormone, and testosterone in the rat. Endocrinology.

[62] Bieglmayer C., Spona J., Adamiker D., Jettmar W. (1980). Basal and LH-RH-stimulated gonadotropin release after transport stress in male rats. Endokrinologie.

[63] McCosh R.B., Breen K.M., Kauffman A.S. (2019). Neural and endocrine mechanisms underlying stress-induced suppression of pulsatile LH secretion. Mol. Cell. Endocrinol..

[64] Alwan M.S., Al-Okialy B.N. (2019). Effect of alpha Lipoic Acid on Some Reproductive Hormones in Adult Male Wister Rats Exposed to Hydrogen Peroxide. Kufa J. Vet. Med. Sci..

[65] Banihani S.A. (2024). Role of Lipoic Acid in Testosterone Production in Males. World J. Men’s Health.

[66] Pollard I., Bassett J., Joss J.M. (1980). Plasma testosterone levels and Δ5-3β-hydroxysteroid dehydrogenase activity in the testis of the rat following prolonged exposure to stress. Reproduction.

[67] Zufry H., Rudijanto A., Soeatmadji D.W., Sakti S.P., Munadi K., Sujuti H., Mintaroem K. (2023). Effects of mobile phone electromagnetic radiation on thyroid glands and hormones in Rattus norvegicus brain: An analysis of thyroid function, reactive oxygen species, and monocarboxylate transporter 8. J. Adv. Pharm. Technol. Res..

[68] Bektas H., Bese Akgun B.B., Cakir S., Dogu S., Ahnas B. (2025). Protective effects of quercetin against 3.5 GHz RF radiation-induced thyroid dysfunction and oxidative stress in rats. Electromagn. Biol. Med..

[69] Koyu A., Cesur G., Ozguner F., Akdogan M., Mollaoglu H., Ozen S. (2005). Effects of 900 MHz electromagnetic field on TSH and thyroid hormones in rats. Toxicol. Lett..

[70] Oyewopo A., Olaniyi S., Oyewopo C., Jimoh A. (2017). Radiofrequency electromagnetic radiation from cell phone causes defective testicular function in male Wistar rats. Andrologia.

[71] Meo S.A., Al-Drees A.M., Husain S., Khan M.M., Imran M.B. (2010). Effects of mobile phone radiation on serum testosterone in Wistar albino rats. Saudi Med. J..

[72] Kesari K.K., Behari J. (2010). Effects of microwave at 2.45 GHz radiations on reproductive system of male rats. Toxicol. Env. Chem..

[73] Kesari K.K., Agarwal A., Henkel R. (2018). Radiations and male fertility. Reprod. Biol. Endocrinol..

[74] Mailankot M., Kunnath A.P., Jayalekshmi H., Koduru B., Valsalan R. (2009). Radio frequency electromagnetic radiation (RF-EMR) from GSM (0.9/1.8 GHz) mobile phones induces oxidative stress and reduces sperm motility in rats. Clinics.

[75] Karadayi A., Akgun Unal N., Gülbahçe Mutlu E., Engiz B., Akkoca A., Varol S. (2023). Effects of Exposure to Radiofrequency at 2.45 GHz on Structural Changes Associated with Lipid Peroxidation in Prepubertal Rat Testicular Tissue. Eur. J. Ther..

[76] Ozorak A., Nazıroğlu M., Celik O., Yüksel M., Ozcelik D., Ozkaya M., Cetin H., Kahya M., Kose A. (2013). Wi-Fi (2.45 GHz)- and Mobile Phone (900 and 1800 MHz)-Induced Risks on Oxidative Stress and Elements in Kidney and Testis of Rats During Pregnancy and the Development of Offspring. Biol. Trace Elem. Res..

