# Supplementary figures and images for: Alpha-Lipoic Acid Preserves Testicular Integrity Under 2.45 GHz Electromagnetic Radiation by Restoring Redox and Inflammatory Balance
Source: Biomedicines. 2025 Dec 15;13(12):3089. doi: 10.3390/biomedicines13123089 (PMC12731164; doi:10.3390/biomedicines13123089)

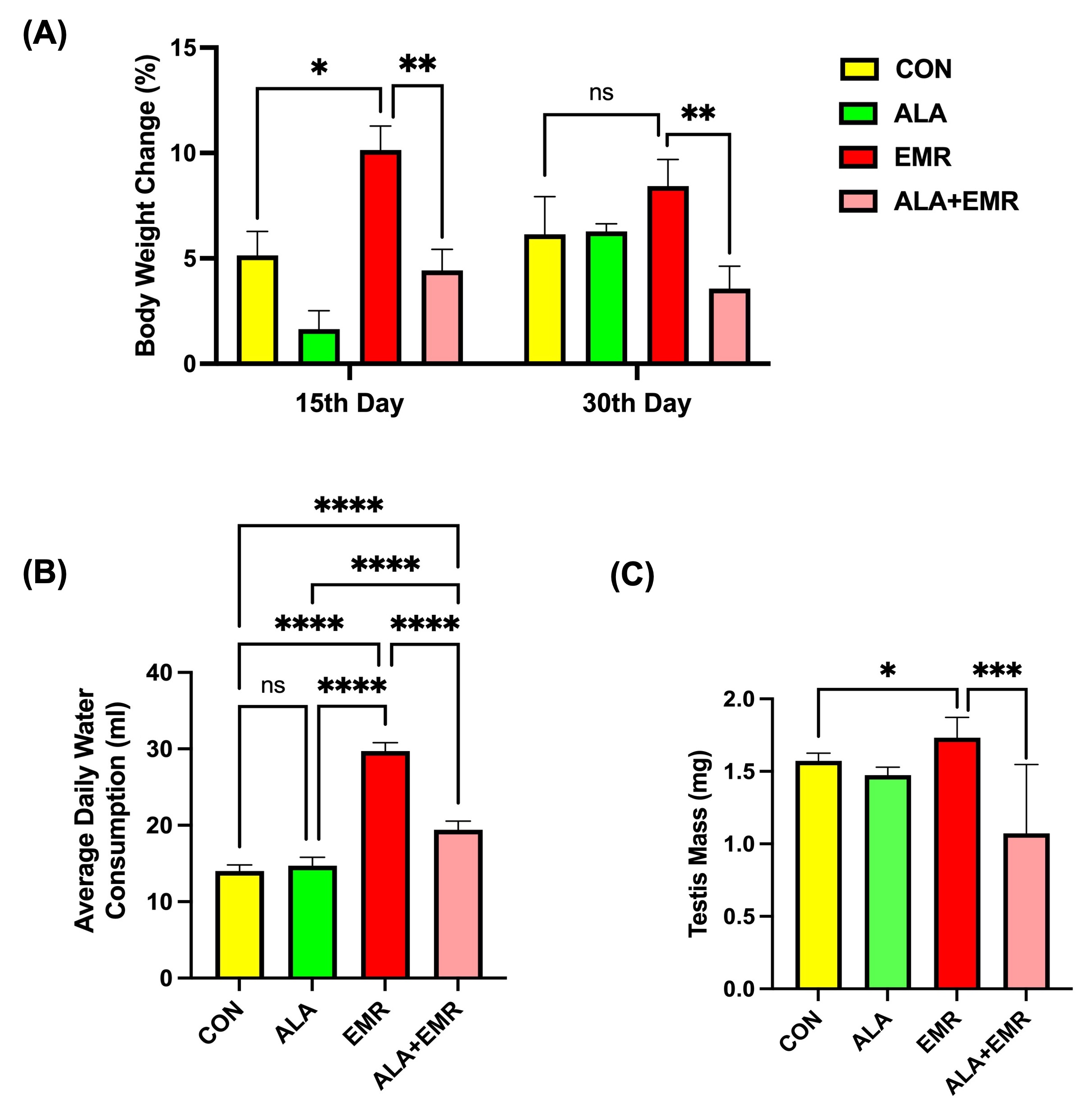

Supplement: Supplementary file 1 [file biomedicines-13-03089-s001.zip › biomedicines-3956311-supplementary.jpg]
